# Biliary Stent Obstruction Leading to Bronchobiliary Fistula: A Rare Case Report

**DOI:** 10.7759/cureus.26514

**Published:** 2022-07-02

**Authors:** Lakshmi Sai Vijay Achalla, Raju K Shinde, Sangita D Jogdand, Anupam Anand, Sahitya Vodithala

**Affiliations:** 1 General Surgery, Datta Meghe Institute of Medical Sciences, Wardha, IND; 2 Pharmacology, Datta Meghe Institute of Medical Sciences, Wardha, IND; 3 Urology, Nil Ratan Sircar Medical College and Hospital, Kolkata, IND; 4 Pathology, Datta Meghe Institute of Medical Sciences, Wardha, IND

**Keywords:** endoscopic retrograde cholangiopancreatography, hepatobiliary tree, hydatid cyst of liver, biliary stent, bronchobiliary fistula

## Abstract

Bronchobiliary fistula (BBF) is a rare complication encountered by surgeons, most commonly during the follow-up of surgically managed patients with inflammatory, traumatic, or neoplastic pathologies involving the hepatobiliary tree. We present an operated case of liver hydatidosis with biliary stent obstruction with complaints of bitter green colored sputum and upper abdominal pain. The patient underwent an ERCP-guided stent extraction with reinsertion of a common bile duct stent with complete removal after six weeks. Post operatively, the patient is doing well on follow-up. This complication could be prevented by regular follow-up and timely removal of the placed stents, as a prolonged stay of stent insitu could lead to blockage, leading to complications such as bronchobiliary fistula. Thus, it is concluded that judicious follow-up plays a pivotal role, and timely removal of the stents could prevent such avoidable delayed complications.

## Introduction

Bronchobiliary fistula (BBF) is a rare complication, and Peacock described it in 1850 [1]. The presence of bile in the sputum and the flow of bile via the bronchi are considered common symptoms (biliptysis). BBF can be congenital or develop after a thoracoabdominal injury. The biliary tract and bronchial trees are abnormally connected [2].

Unlike congenital, acquired BBF is uncommon but typically caused by a local infection, trauma, biliary tract obstruction, or tumor [1]. Biliptysis is a pathognomonic indication of a BBF. BBF is extremely difficult to diagnose, requiring a high clinical index of suspicion [3]. Hepatic, subphrenic abscesses and hepatic malignancies account for the majority of BBFs. Lung and subphrenic abscess, post-operative stenosis (including biliary/gastric or pancreatic surgery), amebic and echinococcosis pyogenic liver abscess, and traumatic/surgical injury to the hepatobiliary system leading to bile flow obstruction are some of the other causes of BBF [2]. BBF has been documented as a post-surgical complication in various cancers, including cholangiocarcinoma, gall bladder carcinoma, and colorectal carcinoma [2]. Most literature describes it as a hydatid disease consequence or a congenital pathology [4]. 

Clinical history and imaging testing are the most popular ways to diagnose it. However, this illness can be difficult to control and is frequently associated with a high morbidity and mortality rate. There has not been a widely approved management strategy earlier [1]. Endoscopic or surgical treatment are generally the options. Here, we present a case of biliary stent blockage as the underlying cause of BBF two years following surgery for hepatic hydatidosis.

## Case presentation

A 32-year-old lady presented with a history of cough followed by a large amount of bitter green colored sputum and mild upper abdominal pain. On physical examination, the patient had decreased breath sounds on the right side with no added sounds. The abdomen was soft with mild tenderness in the right hypochondrium, and there was no guarding/rigidity or distension. The patient had undergone hydatid cyst excision in segment VIII of the liver in 2019. She underwent enucleation of a cyst with omentoplasty. In the post-operative period, the patient developed a persistent biliary leak, evident through the abdominal drain, for which endoscopic biliary stenting was done. A standard plastic biliary stent was used, and the patient was advised for stent removal after three months. However, the patient did not show up and came after two years with these complaints. A complicated lung hydatid with residual hepatic hydatid cyst was suspected clinically.

**Figure 1:**
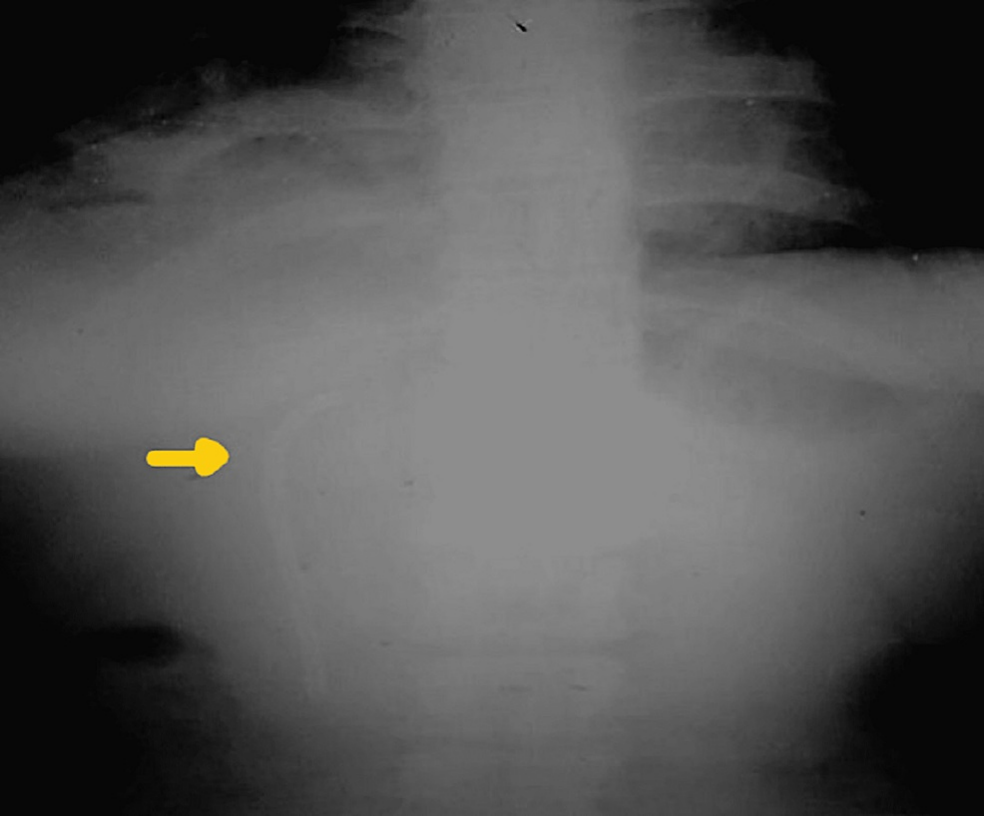
X-ray of the erect abdomen showing the CBD stent insitu. CBD- Common bile duct

**Figure 2:**
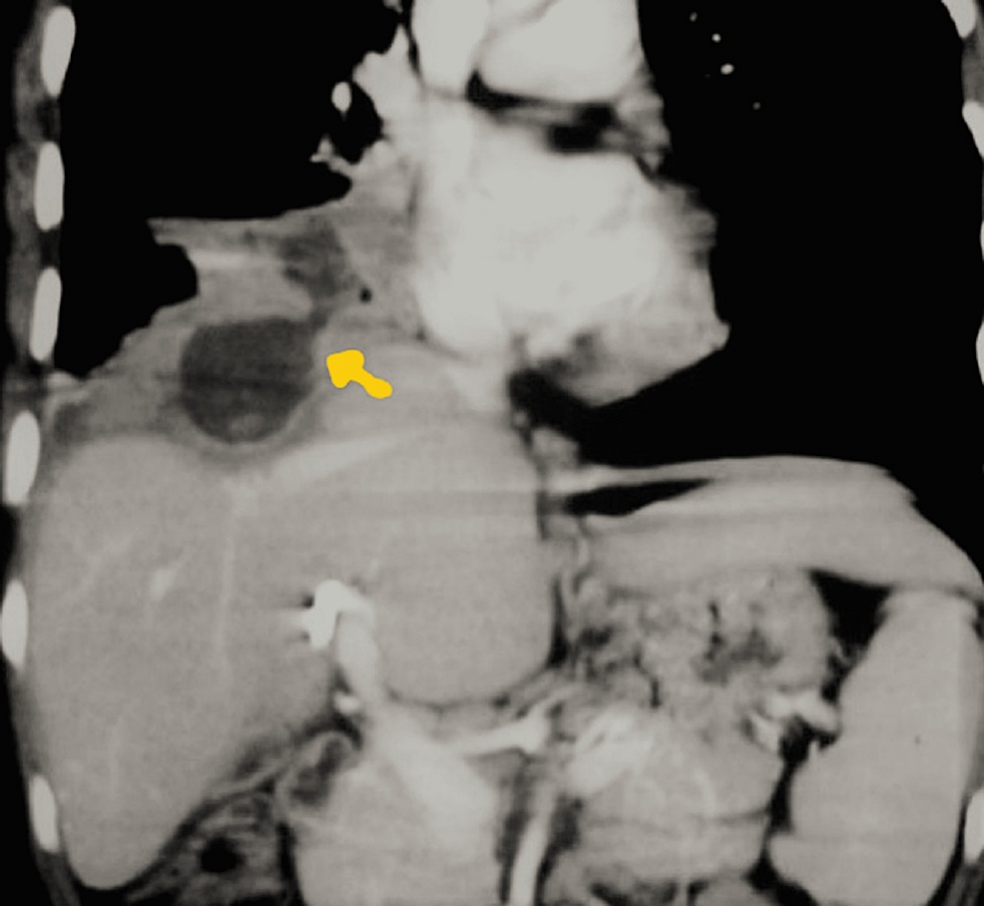
CT abdomen showing bronchobilliary fistula.

Further diagnostic assessments were done, and an X-ray of the erect abdomen showed the placement of a stent ( Figure 1). CT scan abdomen revealed a blocked lumen of the previously placed biliary stent and the presence of communication with the pleural cavity and bronchus to an infra-diaphragmatic area with the consolidation of the right lower lobe of the lung (Figure 2). The biochemical investigations revealed mild conjugated hyperbilirubinemia and increased glutamic-oxalacetic transaminase (SGOT), glutamic pyruvic transaminase (SGPT), and alkaline phosphate levels. Biochemical examination of sputum confirmed the presence of bile in it. Hence the provisional clinical diagnosis of lung hydatidosis or recurrence of hydatid cyst of the liver was ruled out, and a rare complication of an obstructed biliary stent, bronchobiliary fistula, was made.

The patient was subjected to the removed degraded stent by endoscopic retrograde cholangiopancreatography (ERCP) balloon extraction with reinsertion of common bile duct (CBD) stent under antibiotics cover. After six weeks, the biliary stent was removed, and the patient was free of symptoms.

## Discussion

BBF is essentially an abnormal communication between any part of the bronchus and hepatic and extrahepatic biliary tree. The most common cause of BBF is primary tumors of the liver or biliary tree [1]. Other causes could be inflammatory conditions and iatrogenic trauma to a biliary tree in post-operative surgery, resulting in the obstruction of bile flow [5]. BBF can also occur as a complication of liver hydatidosis [6].

 Although BBF has been documented as a congenital abnormality, it is more commonly associated with liver pathology, particularly parasite infections. An inflammatory reaction in the subdiaphragmatic area, subsequent ruptures into the bronchial system, and liver pathology erode the diaphragm, leading to a connection between the bronchial tree and biliary channels, are two mechanisms proposed for the development of BBF. Although hydatid illness is still one of the most common causes of BBF, accounting for 12%, other causes account for 20% of all cases. In recent years, significant surgical interventions and invasive procedures for liver diseases have resulted in these situations [5].

The most common symptom of BBF is biliptysis, i.e., bile in sputum. In sputum analysis, direct and indirect bilirubin levels are detected, indicating communication between the bronchial and biliary trees [5].

In clinical practice, BBF is most usually caused by diagnostic or therapy operations such as hepatobiliary surgery, radiofrequency ablation of liver tumors, ERCPs, and percutaneous transhepatic cholangiography (PTCs) [1,7]. There is currently no specific imaging diagnostic sign for BBF, and biliptyisis is the pathognomonic sign. Invasive procedures such as cholangiography or surgical exploration are used to diagnose BBF. Poor puncture site selection or misoperation can result in iatrogenic BBF [8].

ERCP is ideal for demonstrating the fistulous tract and therapeutic interventions like biliary stricture dilation, stent insertion, and sphincterotomy. Other advanced investigations like contrast-enhanced MRCP can also be done [5]. The techniques currently available include nasobiliary drainage or endoscopic sphincterotomy with biliary endoprostheses. These procedures greatly treat post-operative and posttraumatic biliary leaks [9-12], and they are the first line of treatment in these cases.

The fundamental goal of these endoscopic procedures for bile leaks or fistulas is to eliminate the pressure gradient across the sphincter of Oddi, allowing for preferential bile flow into the duodenum and healing of the leak [13]. BBFs have been successfully treated with nasobiliary drainage [14-16] or endoscopic sphincterotomy with or without stenting [17]. To eliminate the pressure gradient across the sphincter of Oddi and facilitate biliary outflow, endoscopic sphincterotomy alone or in conjunction with papillotomy and stenting may be performed. Post ERCP pancreatitis, bleeding, and duodenal perforation are all possible consequences of the operation [18].

 However, treatment options depend on the primary illness, causative factors, and initial assessment findings. Therapeutic ERCP in the form of stent placement or sphincterotomy can be done. It helps significantly reduce the amount of bile draining into the bronchial system and preserves the lung parenchyma and its functional capacity [1,19]. Surgical intervention is recommended in patients with biliary flow obstruction due to benign diseases, such as lithiasis or hydatid cyst, because of its higher success rate than less invasive endoscopic procedures [20].

## Conclusions

As broncho-biliary fistula is a rare disorder, and several causes can lead to fistula formation, it is important to identify the underlying condition and treat the primary cause. Because of the low prevalence of BBF, there is still no clear consensus on how to treat this uncommon condition. The underlying disease that leads to this uncommon consequence should be considered in the multidisciplinary therapy of such patients. Conservative treatment should be tried initially, followed by endoscopic interventions and surgical resection of the BBF as a last resort if all other options fail. However, as one of the causes of BBF is biliary obstruction due to delayed removal of the biliary stent, timely diagnosis and removal of a stent are highly recommended.
